# Low-grade osteosarcoma after radiotherapy for breast cancer: a case report and literature review

**DOI:** 10.3389/fonc.2026.1758269

**Published:** 2026-03-19

**Authors:** Yajing Sun

**Affiliations:** Department of Pathology, Tianjin Fifth Central Hospital, Tianjin, China

**Keywords:** breast neoplasms, case report, misdiagnosis, neoplasms, post-radiotherapy, osteosarcoma, underdiagnosis

## Abstract

**Objective:**

To investigate the clinicopathological characteristics, diagnostic strategies and clinical management of primary low-grade osteosarcoma of the breast occurring after radiotherapy for breast cancer.

**Methods:**

We report a rare case of primary low-grade osteosarcoma of the breast in a 69-year-old female patient with a history of breast cancer radiotherapy, and review the relevant literature to summarize its clinicopathological features, aiming to improve clinicians’ and pathologists’ recognition and diagnostic capability for this rare disease.

**Results:**

The patient was diagnosed with left breast invasive ductal carcinoma (pT2N0M0, Luminal A type) 12 years ago and underwent breast-conserving surgery followed by postoperative radiotherapy and adjuvant chemotherapy. She presented with a recurrent mass at the original radiotherapy site. Following mastectomy and comprehensive pathological evaluation, the final diagnosis was primary low-grade osteosarcoma of the breast. She then underwent a modified radical mastectomy and has been followed up for 22 months with no signs of local recurrence or distant metastasis to date. Primary osteosarcoma of the breast is rare, typically high-grade, and is associated with a poor prognosis and high recurrence rate. Primary low-grade osteosarcoma is exceptionally rare, with only sporadic case reports suggesting a potentially more favorable prognosis compared to its high-grade counterpart. Its bland histological appearance can lead to misdiagnosis or underdiagnosis, and no standardized treatment protocols have been established for this disease.

**Conclusion:**

Core needle biopsy of radiation-induced low-grade osteosarcoma of the breast is prone to misdiagnosis due to limited sampling and bland histological morphology; comprehensive pathological evaluation of surgical specimens combined with a specific immunohistochemical panel (SATB2, MDM2, β-catenin) is key to a definitive diagnosis. Mastectomy is a reasonable primary treatment option for this rare tumor, and long-term standardized follow-up is necessary. This case report supplements the clinical and pathological data on radiation-induced low-grade osteosarcoma of the breast, and provides a reference for the diagnosis and treatment of this disease.

## 1 Introduction

Osteosarcoma is the most common primary malignant bone tumor originating from osteoblasts, accounting for approximately 20% of all primary bone malignancies. It can be classified into skeletal and extraskeletal subtypes, with extraskeletal osteosarcoma accounting for only 1%~4% of all osteosarcoma cases and occurring in soft tissues without involvement of the contiguous skeletal system. Primary osteosarcoma of the breast is a rare subtype of extraskeletal osteosarcoma, a malignant tumor derived from the non-epithelial components of the breast, composed of spindle cells that produce osteoid stroma and/or bone tissue, and may be accompanied by cartilaginous tissue. It is a rare malignancy, accounting for less than 1% of all mammary malignancies (1, 2). The majority of these tumors are high-grade with an aggressive clinical course and poor prognosis (3, 4). A known risk factor is a history of prior radiotherapy to the breast (1, 4). We present an exceptionally rare case of primary low-grade osteosarcoma of the breast occurring in a patient 12 years after radiotherapy for left breast invasive ductal carcinoma. This case highlights the diagnostic challenges posed by its deceptively bland morphology and adds to the limited literature on this specific entity.

## 2 Case presentation

### 2.1 Baseline clinical information of initial breast cancer

A 69-year-old female patient was admitted to our hospital due to a recurrent mass in the left breast in March 2024. Her medical history showed that she was diagnosed with left breast invasive ductal carcinoma (pT2N0M0, Luminal A type: ER+, PR+, HER2-, Ki-67 8%) at the age of 57 (2012). She underwent breast-conserving surgery for the left breast tumor, followed by postoperative adjuvant radiotherapy and chemotherapy. The radiotherapy parameters were as follows:the irradiation field covered the left breast and regional lymph node drainage area, total dose 50 Gy/25 fractions, 2 Gy per fraction, once daily, 5 fractions a week. The adjuvant chemotherapy regimen was cyclophosphamide + methotrexate + 5-fluorouracil (CMF), 6 cycles in total, and the patient completed the full course of treatment with good compliance. No endocrine therapy was administered due to the patient’s refusal.

### 2.2 Clinical course of the recurrent mass

In 2022 (when the patient was 65 years old), a painless mass was found in the left breast at the site of the original radiotherapy during a physical examination. She underwent local excision (McMoton procedure) in a local hospital. The pathological report indicated reactive fibroblastic hyperplasia, and no further treatment was given. In March 2024, the patient discovered a recurrent mass at the same site that slowly enlarged. She reported no accompanying symptoms such as nipple discharge, breast pain or skin retraction were noted.

### 2.3 Physical examination and imaging findings

On physical examination, a 5 × 3 cm, firm, well-defined, mobile mass was palpated in the left breast at the 2 o’clock position, 5 cm from the nipple, with no tenderness, skin redness or lymphadenopathy in the axillary and supraclavicular fossa.

**Figure 1:**
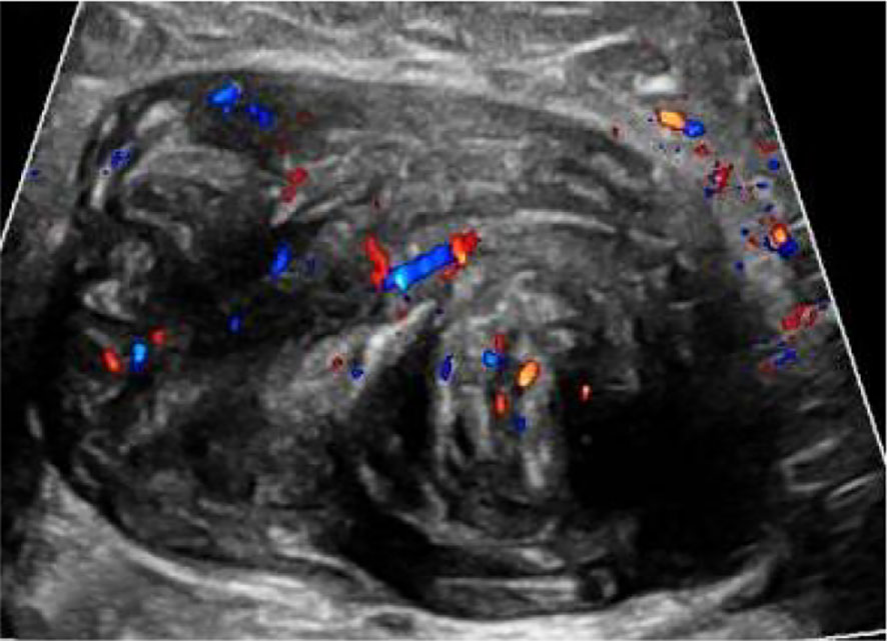
Breast ultrasound showed an irregular hypoechoic solid mass.

**Figure 2:**
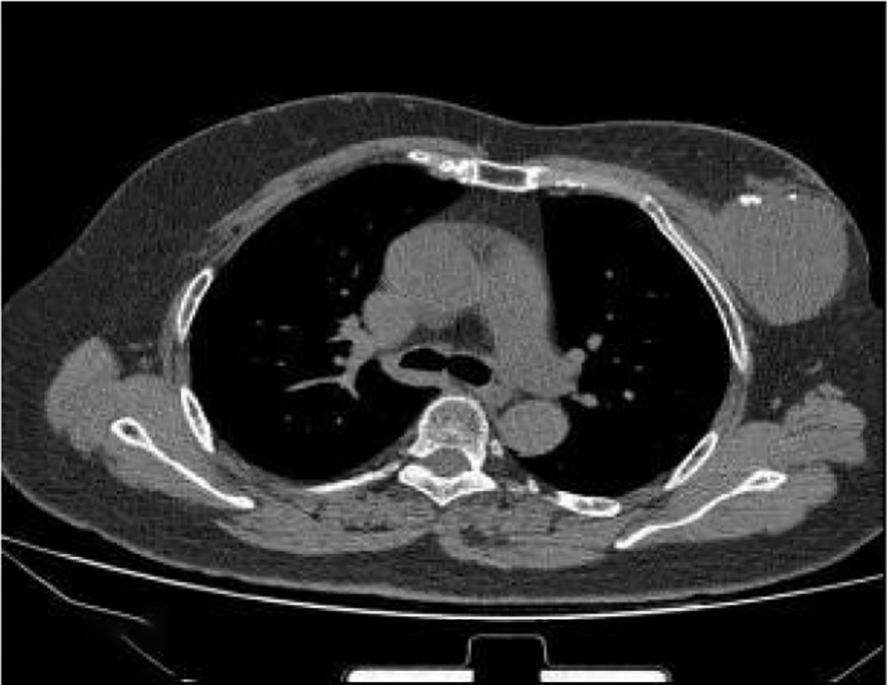
Magnetic resonance imaging revealed a round mass with smooth margins and internal punctate dense shadows.

Breast Ultrasound (US, February 2024, **Figure 1**): According to the ACR BI-RADS Lexicon, the left breast at 2 o’clock position (5 cm from the nipple) showed an irregular hypoechoic solid mass with angular margins, heterogeneous internal echotexture, and abundant internal and peripheral blood flow signals (Type III blood flow), with mild posterior acoustic shadowing. The maximum diameter of the mass was 5.2 cm × 3.1 cm. There was no associated skin thickening or nipple retraction, no enlarged axillary lymph nodes with abnormal morphology. The mass was classified as BI-RADS 4a. Magnetic Resonance Imaging (MRI) revealed a round mass with smooth margins and internal punctate dense shadows (CT value approx. 38 HU), with no obvious enhancement of the surrounding breast tissue and no enlarged lymph nodes in the axilla (**Figure 2**). Tumor marker detection (CA15-3, CEA, CA125) showed all indicators within the normal range.

### 2.4 Preoperative pathological examination (core needle biopsy)

**Figure 3:**
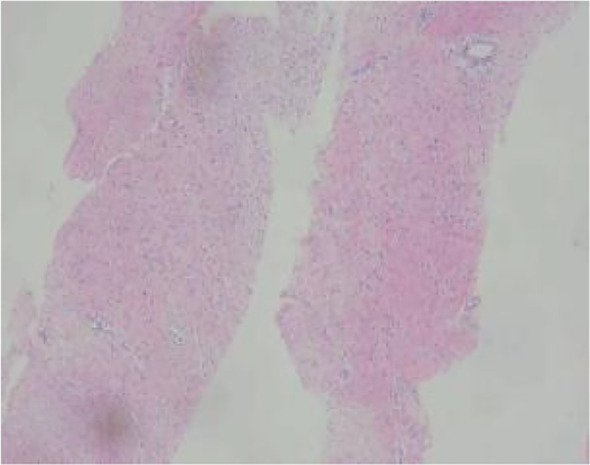
(Low-power H&E) spindle cells arranged in fascicles.

**Figure 4:**
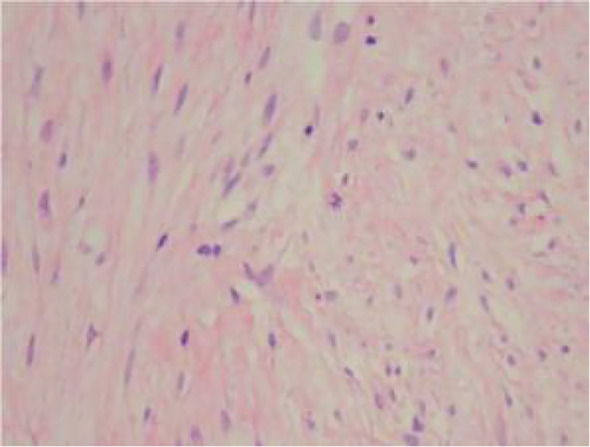
(Medium-power H&E) bland cytomorphology with occasional mitotic figures (arrow).

An ultrasound-guided core needle biopsy was performed for the left breast mass in March 2024. Histological examination revealed a spindle cell proliferation arranged in fascicles and interlacing patterns (**Figure 3**). The cells exhibited low to moderate cellularity with bland morphology, although focal mild atypia and occasional mitotic figures, including atypical forms, were observed (**Figure 4**).

**Figure 5:**
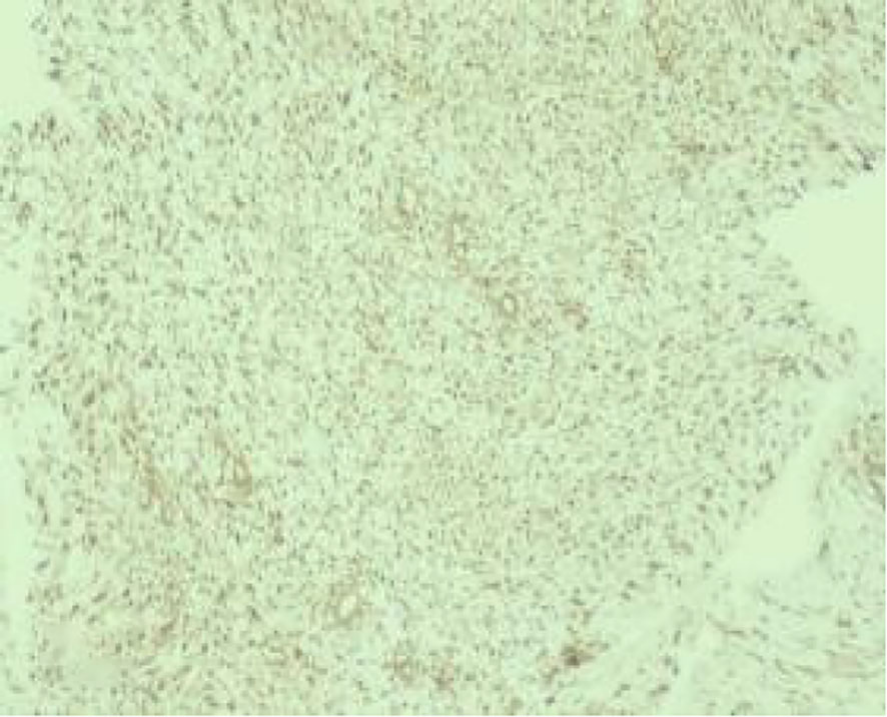
IHC showing diffuse vimentin positivity.

**Figure 6:**
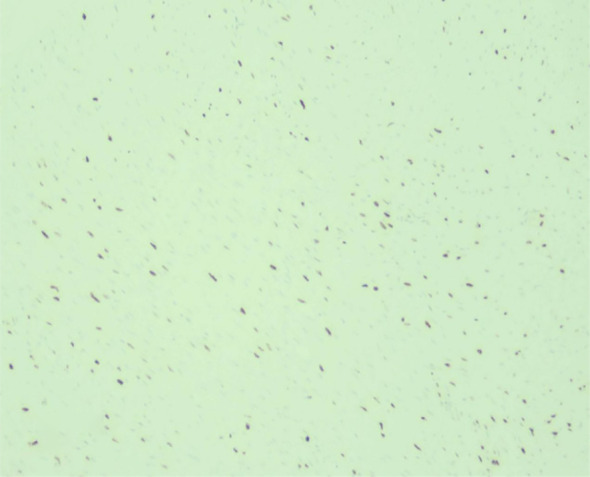
IHC showing Ki-67 positivity (~20%).

Immunohistochemical (IHC) staining was positive for Vimentin (**Figure 5**) and demonstrated a Ki-67 proliferation index of approximately 20% (**Figure 6**). The tumor cells were negative for a broad panel of epithelial markers, including CK, P63, CK5/6, SOX-10, CK7, EMA, and CK34βE12. They were also negative for S100, SMA, STAT6, ER, Desmin, and ALK. HER2 IHC yielded a score of 0. Additionally, the tumor cells showed positive cytoplasmic staining for β-catenin. Based on these findings, the initial pathological diagnosis was: low-grade spindle cell lesion, borderline/low-grade malignant, surgical excision was recommended for definitive diagnosis.

### 2.5 Surgical treatment and treatment rationale

After a multidisciplinary team (MDT) discussion involving pathology, breast surgery, oncology and radiology specialists, a left modified radical mastectomy was selected as the surgical treatment for the patient, and axillary lymph node dissection was not performed (no enlarged axillary lymph nodes were detected on imaging).

Rationale for treatment selection: (1) The tumor was a recurrent spindle cell lesion at the radiotherapy site, with borderline/low-grade malignant features on core needle biopsy, and the actual tumor size may be larger than that indicated by imaging; (2) Breast-conserving surgery was excluded due to the risk of incomplete resection and local recurrence at the previously irradiated site; (3) Adjuvant chemotherapy/radiotherapy was not recommended preoperatively due to the unclear pathological type and the low-grade biological characteristics suggested by the biopsy; (4) The patient, an elderly female with no desire for breast preservation, agreed to mastectomy after thorough informed discussion.

The surgery was performed smoothly in April 2024 with no perioperative complications, and the patient was discharged on the 7th postoperative day.

### 2.6 Postoperative pathological examination

**Figure 7:**
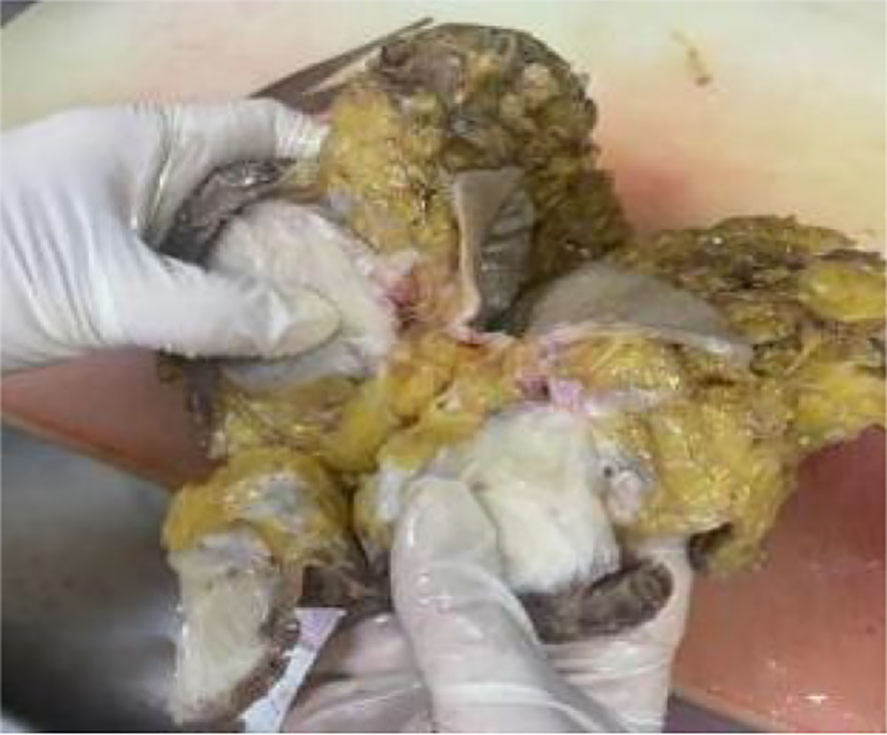
Gross specimen showing a gray-white, solid mass with mostly clear borders and focal invasion.

Gross examination of the mastectomy specimen revealed a circumscribed, firm, gray-white mass measuring 7.2 × 7 × 5 cm, situated 6 cm from the nipple, with focal areas of infiltration into adjacent breast tissue (**Figure 7**). No obvious invasion of the skin or chest wall was found, and no enlarged lymph nodes were found in the peritumoral tissue.

**Figure 8:**
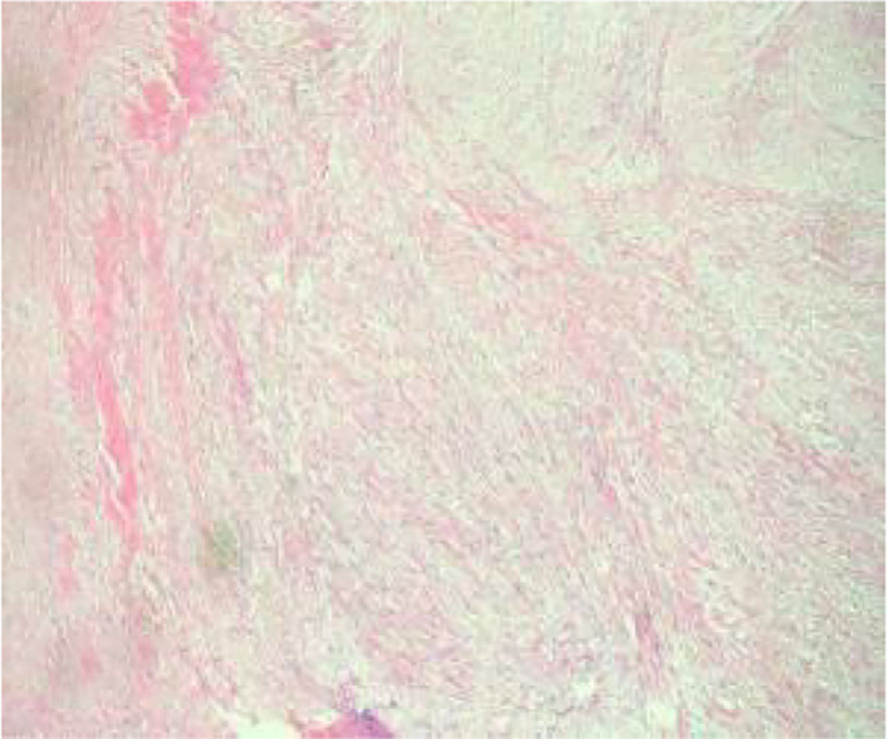
(Low-power H&E) Spindle cells in fascicular, storiform, and woven patterns.

**Figure 9:**
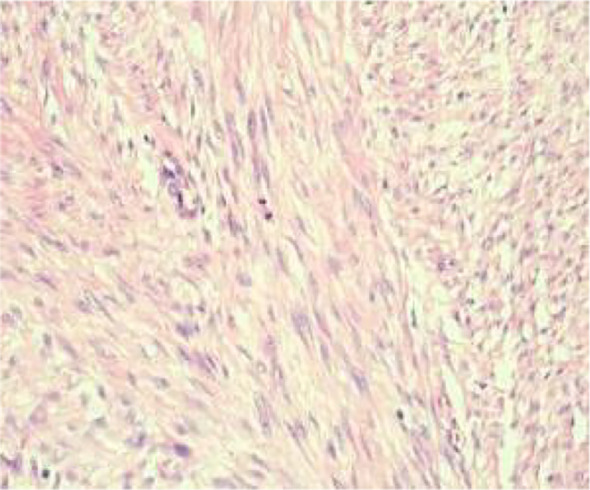
(High-power H&E) Bland tumor cells with a mitotic figure (arrow).

**Figure 10:**
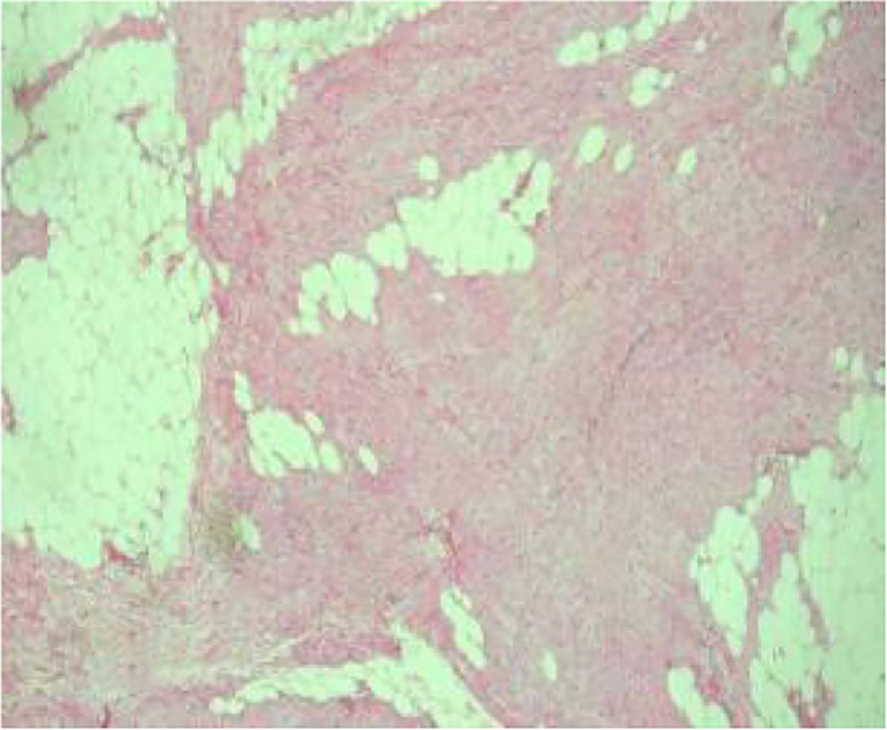
(Low-power H&E) Tumor infiltration into adipose tissue.

**Figure 11:**
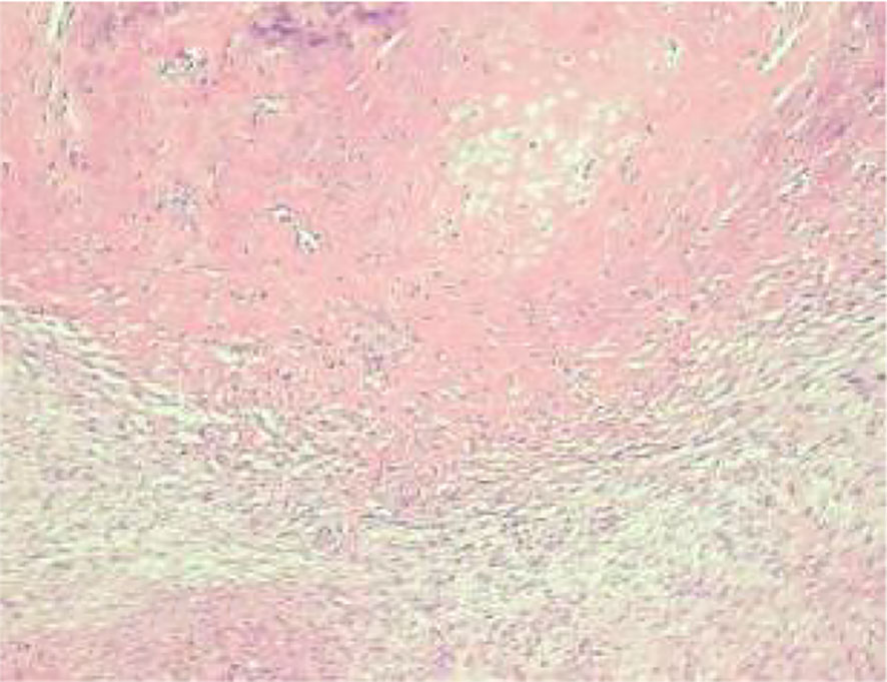
(Medium-power H&E) Chondroid and osteoid matrix.

**Figure 12:**
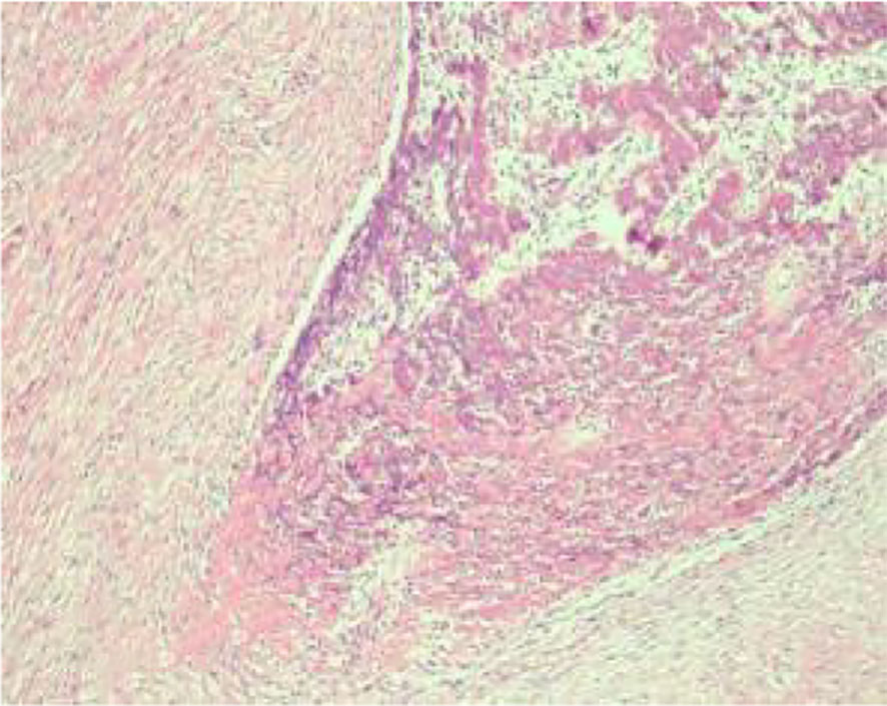
(Medium-power H&E) Tumor bone formation.

Histologic evaluation demonstrated a neoplasm composed of densely packed spindle cells arranged in intersecting fascicles with storiform and woven architectures (**Figure 8**). The tumor cells were predominantly cytologically low-grade; however, peripheral zones exhibited increased cellular density, mild nuclear atypia, and occasional atypical mitotic figures. Infiltration into peritumoral adipose tissue was clearly evident (**Figures 9**, **10**). Significantly, multifocal zones of chondroid and osseous differentiation were identified (**Figures 11**, **12**), with neoplastic bone deposition forming irregular trabeculae and woven patterns—this represented the key pathological feature for diagnosing osteosarcoma. Comprehensive histologic sampling showed no evidence of leaf-like structures or epithelial components, thus ruling out metaplastic carcinoma and phyllodes tumor.

**Figure 13:**
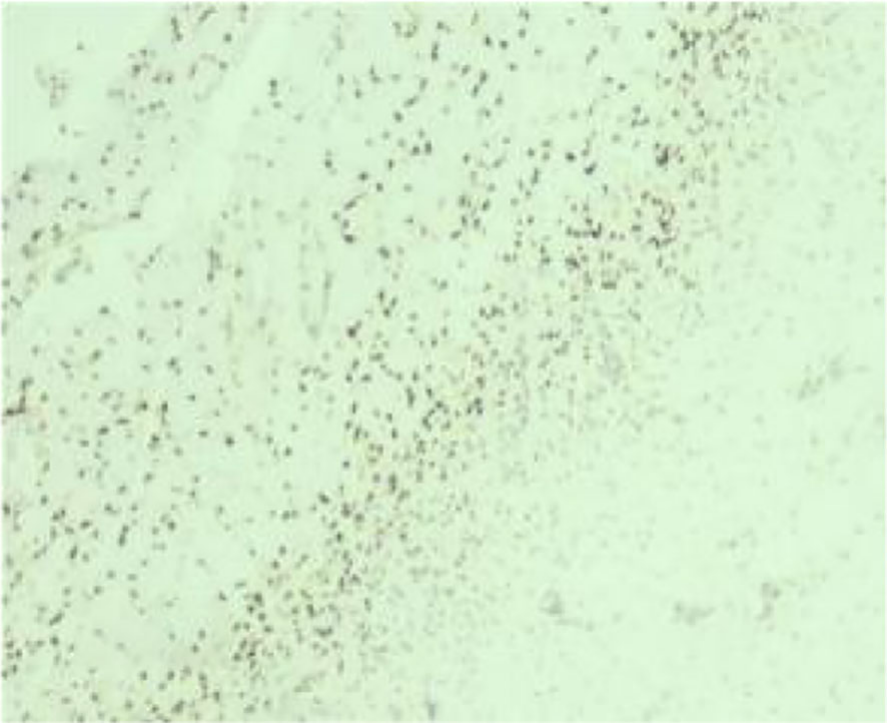
IHC for SATB2 (positive in osteogenic areas).

**Figure 14:**
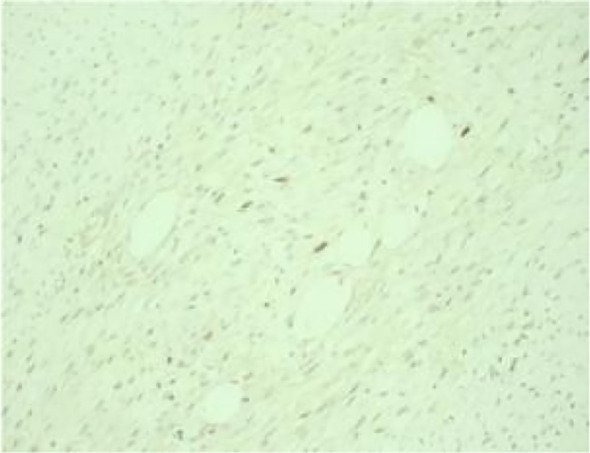
IHC for SATB2 (positive in spindle cells).

**Figure 15:**
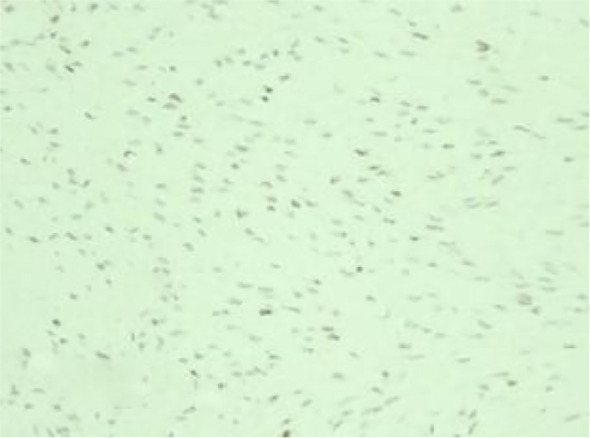
IHC for MDM2 (positive).

**Figure 16:**
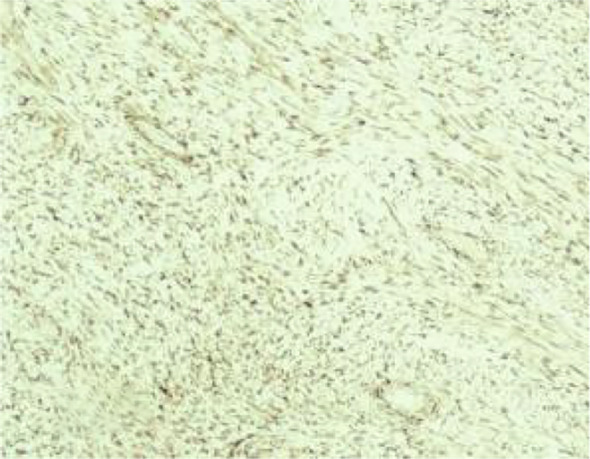
IHC for CD10 (positive).

**Figure 17:**
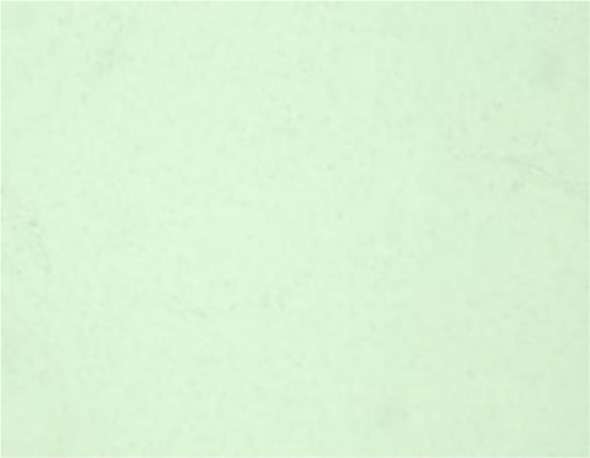
IHC for CK (negative).

**Figure 18:**
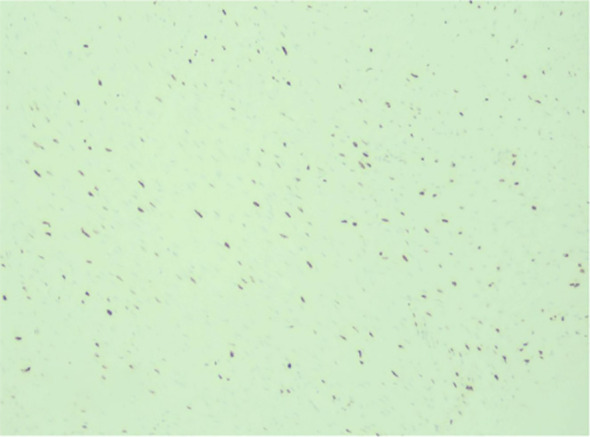
IHC for Ki-67 (~40% in hotspot).

IHC results showed positivity for Vimentin, SATB2 (positive in both osteogenic areas and spindle cells, **Figures 13**, **14**), MDM2 (**Figure 15**), CD10 (**Figure 16**), and CD56. CD34 was locally weakly positive. The tumor cells were negative for CK (**Figure 17**), MUC4, DOG-1, P63, CK5/6, SOX-10, EMA, CK34βE12, S100, SMA, STAT6, and Desmin. β-catenin showed cytoplasmic positivity. The Ki-67 proliferation index ranged from 5% to 40% (hotspot ~40%, **Figure 18**).

Comprehensive systemic workup revealed no evidence of other primary tumors or distant metastases. The final pathological diagnosis was: (Left Breast) Well-Differentiated Osteosarcoma (Low-Grade).

### 2.7 Postoperative follow-up

The patient has undergone standardized follow-up for 22 months after surgery (up to February 2026), with the follow-up schedule including clinical physical examination and breast ultrasound every 3 months, breast MRI every 6 months, and chest/abdominal CT plus tumor marker detection every 12 months. All follow-up assessments showed no local recurrence in the breast or chest wall, no enlarged lymph nodes in the axilla/supraclavicular fossa, and no distant metastasis (lung, bone, liver, etc.). The patient’s general condition was good, with no obvious adverse reactions related to the surgery.

### 2.8 Patient perspective

The patient reported feeling anxious and worried upon finding the recurrent breast mass, particularly due to her history of breast cancer, and she feared tumor recurrence and malignant transformation. During the diagnosis and treatment process, she actively communicated with the medical team, and her anxiety was relieved after the MDT team explained the disease characteristics and treatment plan in detail. She fully agreed to the mastectomy plan and actively cooperated with the postoperative follow-up.

### 2.9 Clinical timeline of the patient’s disease course

**Table 1** clearly illustrates the clinical timeline of the patient’s disease course.

**Table 1:** Clinical timeline of the patient’s disease course.

Time node	Key clinical events
2012 (57 years old)	Diagnosed with left breast invasive ductal carcinoma (pT2N0M0, Luminal A type); received breast-conserving surgery + postoperative radiotherapy (50 Gy/25 fractions) + 6 cycles of CMF chemotherapy
2022 (65 years old)	Recurrent mass in the left breast at the radiotherapy site; local excision (McMoton procedure), pathological diagnosis: reactive fibroblastic hyperplasia
March 2024 (67 years old)	Second recurrence of the mass at the same site; breast ultrasound (BI-RADS 4a) + MRI; core needle biopsy, pathological diagnosis: low-grade spindle cell lesion (borderline/low-grade malignant)
April 2024 (67 years old)	MDT discussion; left modified radical mastectomy of the left breast (no axillary lymph node dissection); postoperative pathological diagnosis: left breast low-grade well-differentiated osteosarcoma (pT3N0M0)
April 2024 – April 2026	22 months of standardized follow-up; no local recurrence or distant metastasis

## 3 Discussion

### 3.1 Clinical characteristics of radiation-induced low-grade osteosarcoma of the breast

Primary osteosarcoma of the breast is a diagnosis of exclusion, which requires ruling out contiguous skeletal primary or distant metastasis (1, 3). It predominantly affects elderly women, with some cases associated with a history of radiotherapy, surgery, or trauma (1, 4). Most reported cases are high-grade, carrying a poor prognosis and high recurrence rate, and no universally accepted standard treatment has been established (3–5).

### 3.2 Diagnostic reasoning

#### 3.2.1 Core needle biopsy stage: differential diagnosis of low-grade spindle cell lesions

The present case is remarkable for its low-grade histology, composed of cytologically bland spindle cells, which is extraordinarily rare (6). This bland morphology poses a significant diagnostic pitfall, particularly in limited samples like core needle biopsies, where the diagnostic osteoid/chondroid component might be missed. This lesion needs to be differentiated from a variety of low-grade spindle cell lesions in the breast, including both tumoral and non-tumoral lesions, as well as benign, borderline and malignant lesions (8, 9). The IHC results (Vimentin+, epithelial markers-, SMA-, STAT6-, β-catenin cytoplasmic+) ruled out metaplastic carcinoma(epithelial markers+), fibromatosis (nuclear β-catenin+, SMA+), reactive spindle cell proliferation (SMA+, Desmin+), nodular fasciitis (SMA+), and solitary fibrous tumor (STAT6+), leading to the diagnosis of “low-grade spindle cell lesion (borderline/low-grade malignant)” and the recommendation for surgical excision to achieve a definitive diagnosis. The osteoid/chondroid differentiation—the key diagnostic feature of osteosarcoma—was not found at this stage due to limited sampling on core needle biopsy, which is the main reason a definitive preoperative diagnosis could not be made.

#### 3.2.2 Postoperative definitive diagnosis stage: confirmation of low-grade osteosarcoma and exclusion of differential diagnoses

The postoperative comprehensive pathological examination identified multifocal chondroid and osseous differentiation with neoplastic bone formation—the pathognomonic feature of osteosarcoma, which serves as the core basis for the diagnosis. Combined with the IHC panel results(SATB2+,MDM2+), we finally confirmed the diagnosis of low-grade well-differentiated osteosarcoma.

Low-grade primary osteosarcoma of the breast requires differentiation from the following entities (10): ①Fibromatosis-like metaplastic carcinoma: When encountering a low-grade spindle cell lesion in the breast, fibromatosis-like metaplastic carcinoma must first be excluded. It presents with bland cellular morphology and infiltrative borders, showing similarities to fibromatosis/scars. Tumor cells are arranged in wavy bundles with uneven density, and may be accompanied by varying degrees of fibrosis. Identification of ductal carcinoma *in situ*, combined with immunohistochemical positivity for epithelial markers such as CK, p63, CK5/6, and CK34βE12, positivity for Vimentin, and a triple-negative immunophenotype, aids in the diagnosis. ②Fibromatosis: This lesion consists of bland spindle cells arranged in fascicles that infiltrate the surrounding tissue, often with peripheral lymphocytic infiltration and rare mitotic figures. Immunohistochemistry shows nuclear β-catenin positivity, SMA positivity, CD34 negativity, and negativity for epithelial markers. Mutations in the CTNNB1 or APC genes are frequently present. ③Phyllodes tumor: In addition to spindle cells, these tumors often exhibit leaf-like/cleft-like structures lined by epithelium. Malignant phyllodes tumors may show heterologous differentiation, such as bone or cartilage. The spindle cell component is typically positive for CD34 and BCL-2, negative for MDM2, STAT6, and CD56, and negative for epithelial markers. ④Nodular fasciitis: Typically located in the subcutaneous superficial fascia, this lesion has a short clinical history, relatively well-defined but unencapsulated borders. It is composed of spindle and plump spindle cells arranged in a tissue culture-like pattern, with pale or faintly eosinophilic cytoplasm, fine chromatin, small nucleoli, and mitotic figures (without atypical forms). The stroma is loose or myxoid, featuring microcysts, extravasated red blood cells, and scattered lymphocytes. It may infiltrate surrounding tissues. Immunohistochemically, it is often diffusely and strongly positive for SMA and MSA, may express calponin and CD10, but is negative for CK, ALK, β-catenin, MDM2, and STAT6. Molecular testing shows *MYH9-USP6* gene fusion in most cases. ⑤Reactive spindle cell proliferation: It represents a reparative response of fibroblasts, and patients usually have a history of core needle biopsy or surgery. The lesion is generally <1 cm in size, nodular, non-encapsulated, with an infiltrative margin. It shows prominent spindle cell proliferation, which may exhibit atypia, but mitotic figures are rare. Inflammatory cells, macrophages, and foam cells are often present. Immunohistochemistry is positive for SMA and Desmin, negative for epithelial markers, MDM2, STAT6, and CD56. ⑥Myofibroblastoma: This is a tumor composed of well-circumscribed, bland spindle cells, characteristically associated with thick collagen bundles and adipose tissue; occasionally, cartilaginous or osseous metaplasia may be seen. It has several morphological variants. Immunohistochemistry shows positivity for CD34, Desmin, SMA, ER, and PR, with a low Ki-67 index. Molecular testing often reveals deletion of 13q14 and loss of the RB1 gene. ⑦Inflammatory myofibroblastic tumor: This tumor consists of spindle tumor cells arranged in fascicles or a storiform pattern, mostly showing mild atypia, accompanied by varying degrees of myxoid change and a mixed inflammatory infiltrate. Immunohistochemical ALK positivity or detection of an ALK gene rearrangement is helpful for diagnosis. ⑧Solitary fibrous tumor: Clinically, it usually presents as a slow-growing, painless mass with well-defined borders. The tumor may have a thin true capsule or pseudocapsule, and typically exhibits a firm consistency; myxoid change can be observed in the stroma in some cases. Histologically, it shows a “patternless” architectural pattern, characterized by alternating hypercellular and hypocellular areas. The tumor cells are relatively monotonous in morphology, and mitotic figures are uncommon. Prominent stromal hyalinization and characteristic staghorn-shaped blood vessels are key diagnostic features. Additionally, the growth pattern of tumor cells can vary, including storiform, herringbone, or short fascicular arrangements.​Immunohistochemically, the tumor demonstrates diffuse and strong nuclear positivity for STAT6, along with positivity for CD34 and BCL-2. Molecular testing typically confirms the presence of a NAB2-STAT6 gene fusion, which is a specific molecular hallmark of this tumor. ​⑨Pseudoangiomatous stromal hyperplasia: This benign proliferation of stromal myofibroblasts commonly occurs in premenopausal women. It features pseudovascular, anastomosing slit-like spaces within a stroma showing varying degrees of sclerosis, lined by bland spindle cells. In some cellular cases, the spindle cells may arrange in fascicles. Immunohistochemistry is positive for CD34, but negative for vascular endothelial markers (e.g., CD31, D2-40, ERG) and epithelial markers. ⑩Schwannoma: This tumor is composed of bland spindle cells arranged in alternating Antoni A (cellular) and Antoni B (hypocellular) areas. The cells are slender and wavy, with Verocay bodies often present. Immunohistochemistry shows positivity for S100 and SOX10.

### 3.3 Treatment and prognosis

At present, no standardized treatment protocol has been established for low-grade osteosarcoma of the breast due to its rarity. For high-grade primary osteosarcoma of the breast, the main treatment is radical surgery combined with adjuvant chemotherapy/radiotherapy, but the prognosis is poor (3, 4, 11, 12). For low-grade cases, radical surgical resection is the main treatment option, and the role of adjuvant chemotherapy/radiotherapy is still controversial (6).

In this case, we selected modified radical mastectomy based on MDT discussion, achieving complete resection of the tumor (R0 resection). Adjuvant chemotherapy/radiotherapy was not administered after surgery for the following reasons: (1) the tumor was low-grade with mild atypia and no distant metastasis, suggesting a relatively indolent biological behavior; (2) the patient was an elderly female (67 years old), with potential poor tolerance to chemotherapy/radiotherapy; (3) the tumor was located at the original radiotherapy site, and re-irradiation may increase the risk of tissue damage and secondary tumors. The 22-month follow-up showed no recurrence or metastasis, suggesting that radical surgical resection alone may be sufficient for low-grade osteosarcoma of the breast, and the prognosis is relatively favorable—this is consistent with the limited literature reports (6).

### 3.4 Diagnostic and clinical implications

This case highlights two key clinical implications for the diagnosis and treatment of radiation-induced low-grade osteosarcoma of the breast:(1) The risk of misdiagnosis on core needle biopsy: The bland histological morphology of low-grade osteosarcoma and the lack of osteoid/chondroid differentiation in limited sampling can easily lead to misdiagnosis or underdiagnosis. For low-grade spindle cell lesions of the breast in patients with a history of radiotherapy, pathologists should be alert to the possibility of osteosarcoma, and clinicians should recommend surgical excision for definitive diagnosis when the biopsy result is inconclusive.(2) The importance of a specific IHC panel: A comprehensive IHC panel including SATB2, MDM2, β-catenin, epithelial markers (CK, P63) and mesenchymal markers (SMA, STAT6, S100) is essential for the differential diagnosis of low-grade osteosarcoma of the breast, among which SATB2 is a specific marker for confirming osteogenic differentiation.(3) MDT and standardized follow-up: MDT discussion is helpful for the rational selection of treatment options for this rare tumor, and long-term standardized follow-up is necessary due to the lack of long-term prognostic data.

## 4 Conclusion

Radiation-induced low-grade osteosarcoma of the breast is an extremely rare extraskeletal osteosarcoma subtype, and its core diagnostic challenge lies in the high misdiagnosis rate of core needle biopsy caused by limited sampling and bland histological morphology. The key to a definitive diagnosis is the comprehensive pathological evaluation of surgical specimens, including the identification of pathognomonic osteoid/chondroid differentiation and neoplastic bone formation, combined with a specific immunohistochemical panel, which can effectively exclude other similar low-grade spindle cell lesions of the breast. Furthermore, when an osteosarcomatous component is identified, it is essential to first exclude metaplastic carcinoma and malignant phyllodes tumor before considering a diagnosis of primary osteosarcoma of the breast.

Low-grade osteosarcoma of the breast is an extremely rare radiation-induced secondary malignancy, with a relatively favorable prognosis compared to high-grade osteosarcoma. Radical surgical resection (e.g., mastectomy) is a reasonable primary treatment option for this disease, and the role of adjuvant chemotherapy/radiotherapy needs to be further explored with more clinical data. Long-term standardized follow-up is necessary for all patients due to the lack of standardized treatment protocols and long-term prognostic data.

This case report supplements the detailed clinical, pathological and follow-up data of radiation-induced low-grade osteosarcoma of the breast in accordance with the CARE guidelines, which can improve the recognition of this rare disease among clinicians and pathologists and provide a practical reference for its diagnosis and treatment. More case reports and multi-center studies are needed to accumulate clinical data and formulate standardized diagnosis and treatment protocols for this rare tumor (4, 5, 7).

## Data Availability

The datasets presented in this study can be found in online repositories. The names of the repository/repositories and accession number(s) can be found in the article/supplementary material.
